# Genetic variants of *ERBB4* gene and risk of gestational diabetes mellitus: a susceptibility and diagnostic nomogram study

**DOI:** 10.3389/fendo.2023.1283539

**Published:** 2023-12-12

**Authors:** Ruiqi Li, Yukun Wang, Lin Yang, Ping Zhong, Gongchen Huang, Qiulian Liang, Xiangyuan Yu

**Affiliations:** ^1^ The Guangxi Key Laboratory of Environmental Exposomics and Entire Lifecycle Health, Guilin Medical University, Guilin, China; ^2^ Scientific Experiment Center, Guilin Medical University, Guilin, China; ^3^ Department of Obstetrics and Gynecology, The Second Affiliated Hospital of Guilin Medical University, Guilin, China

**Keywords:** Erb-b2 receptor tyrosine kinase 4, gestational diabetes mellitus, variant, Association, function, nomogram

## Abstract

**Introduction:**

Gestational diabetes (GDM) is one of the common complications of female pregnancy, which seriously affects the health of mothers and their offspring. So far, the etiology has not been fully clarified.

**Methods:**

A case-control study was conducted to clarify the relationship between Erb-b2 receptor tyrosine kinase 4 (ERBB4) functional tag genetic variants (rs1595064, rs1595065, rs1595066 and rs6719645) and the risk of GDM. Associations between variants and GDM risk were evaluated with the odds ratios (ORs) and their corresponding 95% confidence intervals (CIs). Subsequently, the false-positive reporting probability (FPRP), multi-factor dimension reduction (MDR) and bioinformatics analysis were adopted to confirm the significant associations. A nomogram model was constructed to predict the risk of GDM.

**Results:**

Association analysis demonstrated that rs1595066 TT genotype performed a protective effect on GDM risk among all subjects (TT vs. CC: adjusted OR = 0.60, 95% CI = 0.38 - 0.94, *P* = 0.026; TT vs. CC/CT: adjusted OR = 0.61, 95% CI = 0.40 - 0.95, *P* = 0.027). Meanwhile, stratified analysis showed that rs1595066 TT can also reduce the GDM risk in age > 30.09 years old, pre-pregnancy BMI > 22.23 Kg/m^2^, SBP ≤ 110.08 mmHg, etc subgroups. Interactions between rs1595066 and DBP (*P*_interaction_ = 0.01), FPG (*P*_interaction_ < 0.001) and HbA1c (*P*_interaction_ < 0.001) were detected. The FPRP analysis confirmed that association between rs1595066 and GDM risk in subjects of FPG < 4.79 mmol/L (*P* = 0.199) is true. The MDR analysis showed that rs1595066 was the best single locus model while the 4-loci model was the best multiple factors model to predict GDM risk. Functional prediction revealed that rs1595066 may disturb the stability of miRNA-mRNA binding. The predictive nomogram model has a well consistence and acceptable discriminative ability with a diagnosed AUC of 0.813.

**Discussion:**

*ERBB4* variants can change an individual’s susceptibility to GDM via the interaction of gene-gene, gene-environment and changes in the regulatory effects of miRNAs on *ERBB4* expression.

## 1 Introduction

Gestational diabetes mellitus (GDM) is one of the most common metabolic complications in women during pregnancy, affecting approximately 1%–14% of pregnant women worldwide (1, 2). GDM typically manifests in the second and third trimesters of pregnancy. In China, the incidence of GDM is approximately 14.8% (3). GDM may cause serious complications in mothers and infants, such as gestational hypertension, polyhydramnios, spontaneous abortion, preterm birth, respiratory distress syndrome, small or large for gestational age, fetal macrosomia, and even stillbirth (4–6). GDM poses a threat to pregnant women and their offspring.

The main risk factors for GDM are often older age at pregnancy, obesity, family history of type 2 diabetes mellitus (T2DM), history of GDM, and previous poor obstetric history. Studies also support a similar pathological mechanism of insulin resistance and β-cell dysfunction in GDM and T2DM (7). However, the specific underlying causes of GDM have not been fully elucidated. Previous studies have shown that the incidence of GDM is directly proportional to the prevalence of T2DM in different ethnic groups. GDM is particularly common among certain female populations, with Asian, Native American, Hispanic, and African American women at a significantly higher risk of developing GDM than non-Hispanic Caucasians (2). Having a family history of diabetes or past history of GDM is an independent risk factor for pregnancies developing GDM, while offspring of GDM patients have a significantly higher risk of developing T2DM (8–10). The genetic background of an individual plays a crucial role in the development of GDM.

Single-nucleotide polymorphisms (SNPs) are DNA sequence variations caused by a single nucleotide either through conversion or transversion. To the best of our knowledge, SNPs that are significantly associated with diseases can exist at any location in the genome. SNPs located in different functional regions of genes may affect the function of genomic DNA components, mRNA levels produced by transcription, protein translation, and even cause changes in biological traits. SNPs contain crucial information that determines the genetic susceptibility to complex human diseases and have been widely applied in the screening of high-risk populations and disease risk prediction models (11). Currently, a series of GDM susceptibility genes and susceptible SNPs have been identified by candidate gene studies and genome-wide association studies (GWAS) (12–16). These SNPs either affect disease risk or are significantly associated with genetic susceptibility to GDM through interactions with age, pre-pregnancy body mass index (pre-BMI), blood sugar, and blood lipids. Therefore, it is feasible to construct a predictive model based on significantly associated SNPs and key clinical variables to predict the risk of GDM.

The nomogram model constructed based on the logistic regression algorithm can score the value level of each influencing factor involved according to the size of the regression coefficient and finally read the probability of corresponding outcome events by calculating the total score of each individual (17). The Nomogram model can intuitively express the quantitative relationship between independent risk factors in the model and visualize it in a column chart format, effectively predicting the risk of individual GDM occurrence and providing an important reference for personalized GDM prevention and control. In addition, the evaluation of the effectiveness of building a model is an important step in evaluating the fit and applicability of risk prediction models and serves as a basis for further improving the construction and selection of the best model. The evaluation of nomogram prediction models is mainly reflected through indicators such as discrimination and calibration (18). In this study, a logistic regression model was used to evaluate the predictive ability of the model by applying the area under the receiver operating characteristic curve (AUC) to the subjects.

Erb-b2 receptor tyrosine kinase 4 (ERBB4), also known as HER4, is a member of the Tyr protein kinase family and epidermal growth factor receptor subfamily (19). The activation of ErbB receptors occurs by binding to specific ligands, initiating downstream signaling pathways, such as mitogen-activated protein kinase (MAPK), phosphoinositol 3-kinase/protein kinase B (PI3K/AKT), and mammalian rapamycin target (mTOR) signaling pathways, which regulate energy homeostasis (20). In addition, ERBB4 binding to its specific ligand neuregulin 4 (NRG4) improves insulin sensitivity, whereas blocking antibodies against ERBB4 weakens NRG1-induced glucose uptake (21, 22). *ERBB4* and its variants are associated with type 1 or type 2 diabetic nephropathy, polycystic ovary syndrome, obesity, hyperglycemia, hyperinsulinemia, and insulin resistance (23–28). At present, there are no clear studies on the association between *ERBB4* variants and GDM onset.

In this study, four variants (rs1595064, rs1595065, rs1595066, and rs6719645) located at the *ERBB4* gene were genotyped in 554 GDM patients and 641 normal pregnant women, to explore the genetic susceptibility of *ERBB4* variants to GDM. In addition, we constructed a nomogram predictive model based on the identified significantly associated SNPs and key clinical variables for predicting GDM in the early stages of pregnancy.

## 2 Materials and methods

### 2.1 Subject selection

**Figure 1:**
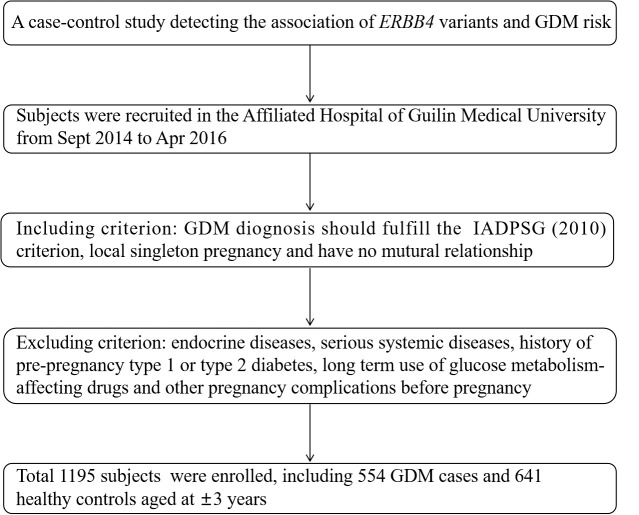
Flowchart of recruitment of subjects in a case–control study.

All subjects were recruited from the Affiliated Hospital of Guilin Medical University in September 2014 to April 2016, including 554 GDM cases with a mean age of 31.55 ± 4.76 years old and 641 healthy pregnancies aged 28.83 ± 4.13 years old. GDM was identified through the diagnosis criteria proposed by the International Association of Diabetes and Pregnancy Study Group (IADPSG) based on at least one changed threshold in following 75 g oral glucose tolerance test (OGTT): fasting plasma glucose (FPG) (≥5.1 mmol/L); 1 hour (1 h) blood glucose (≥10.0 mmol/L); 2 h blood glucose (≥8.5 mmol/L). Included volunteers were required to fulfill the following requirements: have lived in Guilin area for more than 2 years, have no mutual family relationship and is a singleton pregnancy this time. Pregnancies that have developed endocrine diseases, serious systemic diseases, history of pre-pregnancy type 1 or type 2 diabetes, long-term use of drugs affecting glucose metabolism, or other pregnancy complications before pregnancy will be excluded. The screening flowchart of the subjects is shown in **Figure 1**. The Ethics Committee of Guilin Medical University approved this study and all participants provided informed consent.

### 2.2 Demographic and biochemical data collection

Subject information such as age, pre-pregnancy weight, height (pre-pregnancy body mass index (pre-BMI)), systolic blood pressure (SBP), diastolic blood pressure (DBP), hemoglobin A1c (HbA1c), fasting plasma glucose (FPG), 75 g OGTT 1 h and 2 h blood glucose, triglyceride (TG), total cholesterol (TC), low-density lipoprotein (LDL) and high-density lipoprotein (HDL) levels were obtained from the same questionnaires and hospital records.

### 2.3 DNA extraction, candidate variants selection, and genotyping

Genomic DNA was extracted from EDTA-treated whole blood using a DNA extraction kit (Aidlab Biotechnologies Co., Ltd, China) and stored at −80°C prior to PCR.

Based on our previous GWAS, which included 96 cases of GDM and 96 controls, the potential functional variant rs6719645 at *ERBB4* was significantly associated with GDM risk (*P* = 1.133 × 10^−4^). According to this preliminarily clue, the SNP Function Prediction (FuncPred) tool of SNPinfo Web Server (https://manticore.niehs.nih.gov/snpinfo/snpfunc.html) was first used to screen *ERBB4* gene functional variants in the Chinese Han population in Beijing (CHB) with a minimum allele frequency (MAF) >0.1, and 13 variants were initially identified. The LD Tag SNP selection tool of SNPinfo Web Server (https://manticore.niehs.nih.gov/snpinfo/snptag.html) was performed to select the Tag variants with r^2^ >0.8 (29). Finally, five variants (rs6719645, rs1595064, rs1595065, rs1595066, and rs3748962) were selected.

The Sequenom MassARRAY platform was used for candidate variants genotyping. The PCR master mix was composed of 1 μl template DNA (20 ng/μl–100 ng/μl), 1.850 μl ddH_2_O, 0.625 μl of 1.25× PCR buffer (15 mmol/L MgCl_2_), 0.325 μl of 25 mmol/L MgCl_2_, 0.1 μl of 25 mmol/L dNTP mix, 1 μl of 0.5 μmol/L primer mix, and 0.1 μl of 5 U/μl HotStar Taq polymerase. The reaction was conducted at 94°C for 15 min, followed by 45 cycles at 94°C for 20 s, 56°C for 30 s, and 72°C for 1 min, with a final incubation at 72°C for 3 min. The following steps were shrimp alkali enzyme purification (SAP) reaction, single-base extension reaction, resin purification, and chip sampling, respectively. Original data and genotyping plots were obtained using TYPER 4.0.

**Figure 2:**
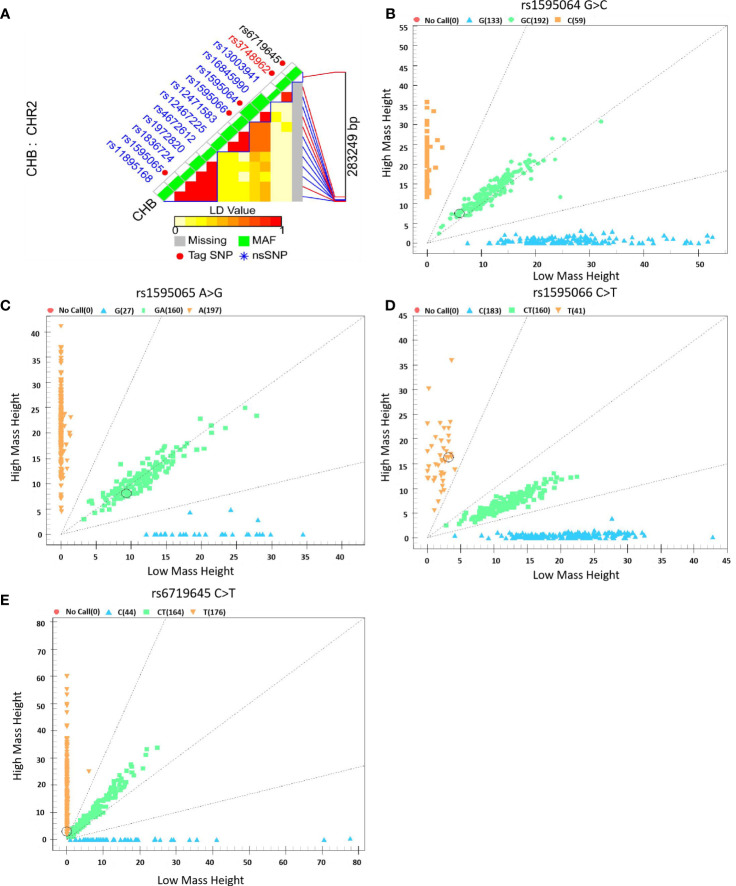
Selection and genotyping of *ERBB4* variants. **(A)** *ERBB4* variant selection using the LD Tag SNP selection tool with a r^2^ >0.8, **(B–E)** Genotyping plots of *ERBB4* variants using the Sequenom MassARRAY platform.

However, owing to the unsuccessful genotyping of rs3748962, it will no longer be analyzed subsequently, as shown in **Figure 2**.

### 2.4 Statistical analysis

Metrological data that conform to a normal distribution are expressed as mean ± standard deviation ( $\bar{\text{x}}$ ± sd), and Student’s t-tests were used for comparison between the two groups, while non-normally distributed data are represented by the median (interquartile range, IQR), and non-parametric tests were used for comparison between the two groups. The chi-square (χ^2^) test was used to compare of categorical variables, and the χ^2^ goodness-of-fit method was used to test whether the genotype frequency distribution followed by the Hardy–Weinberg equilibrium (HWE). Odds ratios (ORs) and their corresponding 95% confidence intervals (CIs) were calculated using binary logistic regression to evaluate the associations between variants and GDM risk. Stratified analysis was also performed to detect the correlation between positive SNP and GDM risk in different subgroups based on the mean value of the variables. Data analysis was performed using IBM SPSS Statistics 28 for Windows (IBM Corp., Armonk, NY, USA), and two-sided test *P<*0.05 was considered statistically significant.

To account for chance associations, false-positive report probability (FPRP) analysis was used to assess false-positive association findings (30). The FPRP cutoff value of 0.2 and a prior probability level of 0.1 were preset to detect an OR of 2 or 0.5, which is most likely to be associated with genotypes. Only *P*_FPRP_ values<0.2, were considered true.

Multifactor dimension reduction (MDR) software (version 3.0.2) was adopted to investigate the interactions between variants with a preset 100-fold cross-validation, the best testing-balanced accuracy (TBA), and cross-validation consistency (CVC) (31). As predicted by the SNPinfo Web Server (https://manticore.niehs.nih.gov/snpinfo/snpfunc.html) (29), three loci of the *ERBB4* gene (rs1595064, rs1595065, and rs1595066) were located at the miRNA binding sites; thus, an appropriate functional analysis was conducted.

The nomogram model can read the probability of the corresponding outcome events of an individual with a total score composed of scores incorporating factors according to the logistic regression coefficient (32, 33). Based on the scoring of the key clinical traits and interacting *ERBB4* variants, a predictive nomogram model was constructed to assess GDM risk in pregnant women. The discriminant ability of the predictive model was evaluated by drawing a receiver operating characteristic (ROC) curve and calculating the area under the curve (AUC). A calibration curve was applied to assess the accuracy of the predictive model and 1,000 bootstrap resamples were performed for internal validation. In addition, decision curve analysis (DCA) was conducted to determine the clinical utility of the model by quantifying the net benefit at different threshold probabilities.

## 3 Results

### 3.1 The characteristic of subjects

The clinical and biochemical data of the patients are presented in **Table 1**. Compared to healthy controls, the levels of GDM cases were higher in age, pre-BMI, and the levels of SBP, DBP, TG, HbA1c, and FPG, 75 g OGTT at 1 h, and 2 h blood glucose (*P<*0.001).

**Table 1:** Demographic and clinical characteristics in cases and controls (mean ± sd).

Clinical traits	GDM (n = 554)	Controls (n = 641)	*t*	*P*
Age (years old)	31.55 ± 4.76	28.83 ± 4.13	10.44	<0.001
Pre-pregnancy BMI (Kg/m^2^)	23.14 ± 3.61	21.44 ± 3.00	8.72	<0.001
SBP (mmHg)	111.61 ± 10.59	108.76 ± 9.38	4.89	<0.001
DBP (mmHg)	70.51 ± 8.74	68.68 ± 7.90	3.78	<0.001
FPG (mmol/L)	5.22 ± 1.33	4.41 ± 0.37	13.96	<0.001
1hPG (mmol/L)	9.76 ± 2.25	6.96 ± 1.43	25.30	<0.001
2hPG (mmol/L)	8.30 ± 2.17	6.08 ± 1.10	21.85	<0.001
HbA1c (%)	5.44 ± 0.68	5.00 ± 0.48	12.62	<0.001
TG (mmol/L)	2.67 ± 1.20	2.43 ± 1.01	3.82	<0.001
TC (mmol/L)	5.37 ± 1.15	5.29 ± 1.07	1.30	0.194
HDL-c (mmol/L)	1.66 ± 0.42	1.65 ± 0.40	0.31	0.756
LDL-c (mmol/L)	3.49 ± 1.02	3.45 ± 1.01	0.68	0.496

SBP, systolic blood pressure; DBP, diastolic blood pressure; FPG, fasting plasma glucose; TG, Triglyceride; TC, Total cholesterol; HDL-c, high density lipoprotein cholesterol.

### 3.2 Association of functional variants and GDM risk

After adjusting for age and pre-BMI, the logistic regression analysis demonstrated that, compared to the CC genotype, rs1595066 TT could significantly decrease GDM risk by 40% (adjusted OR = 0.60, 95% CI = 0.38–0.94, *P* = 0.026). In the recessive model, compared to the CC/CT genotypes, the TT genotype also had a protective effect on individual susceptibility to GDM (adjusted OR = 0.61, 95% CI = 0.40–0.95, *P* = 0.027). However, there was no significant association between the rs1595064, rs1595065 and rs6719645 and GDM risk observed, as shown in **Table 2**.

**Table 2:** Association analysis between *ERBB4* SNPs and GDM risk.

Variant, rs^#^	Genotype	Case	Control	*P* ^a^	Adjusted OR (95% CI)	*P* ^b^
rs1595064	GG	197	221	0.921	1	
	GC	264	312		0.94 (0.72–1.23)	0.658
	CC	93	108		0.88 (0.62–1.27)	0.505
	GC/CC	357	420	0.696	0.93 (0.72–1.19)	0.554
	GG/GC	461	533		1	
	CC	93	108	0.977	0.92 (0.66–1.27)	0.598
rs1595065	AA	293	331	0.599	1	
	AG	219	251		0.93 (0.72–1.21)	0.594
	GG	42	59		0.85 (0.53–1.34)	0.474
	AG/GG	261	310	0.666	0.92 (0.72–1.17)	0.486
	AA/AG	512	582		1	
	GG	42	59	0.314	0.87 (0.56–1.36)	0.545
rs1595066	CC	262	303	0.141	1	
	CT	225	255		0.96 (0.74–1.24)	0.733
	TT	42	73		0.60 (0.38–0.94)	**0.026**
	CT/TT	247	328	0.442	0.88 (0.69–1.12)	0.303
	CC/CT	487	558		1	
	TT	42	73	**0.049**	0.61 (0.40–0.95)	**0.027**
rs6719645	CC	65	82	0.466	1	
	CT	230	282		1.02 (0.69–1.51)	0.933
	TT	259	277		1.10 (0.74–1.62)	0.638
	CT/TT	489	559	0.578	1.06 (0.73–1.53)	0.766
	CC/CT	295	364		1	
	TT	259	277	0.220	1.08 (0.85–1.39)	0.518

a, Genotype distribution difference tested by χ^2^; b, Adjusted for age, BMI in logistic regression models. The symbol Rs^#^: Denotes variant variables. Bold values indicate that the differences are statistically significant.

Stratified analysis was applied to evaluate the relationship between rs1595066 and GDM risk using the recessive model. Compared with CC/CT genotypes, TT genotype could significantly reduce the risk of GDM in subjects of age >30.09 years (adjusted OR = 0.45, 95% CI = 0.24–0.84, *P* = 0.013), BMI >22.23 Kg/m^2^ (adjusted OR = 0.52, 95% CI = 0.28–0.98, *P* = 0.042), SBP ≤110.08 mmHg (adjusted OR = 0.55, 95% CI = 0.31–0.98, *P* = 0.043), FPG ≤4.79 mmol/L (adjusted OR = 0.40, 95% CI = 0.20–0.78, *P* = 0.007), 2hPG ≤7.11 mmol/L (adjusted OR = 0.47, 95% CI = 0.23–0.98, *P* = 0.043) and HbA1c ≤5.20% (adjusted OR = 0.51, 95% CI = 0.27–0.97, *P* = 0.040). Surprisingly, significant interactive effects were found between the genetic loci rs1595066 and DBP (*P*_interaction_ = 0.01), FPG (*P*_interaction_ *<*0.001) and HbA1c (*P*_interaction_ *<*0.001) (**Table 3**).

**Table 3:** Stratified analysis of rs1595066 and GDM risk.

Variables^a^	CC/CT (Case/Control)	TT (Case/Control)	Adjusted OR (95%CI)	*P* ^b^	*P* ^c^
Age (years)					0.339
≤30.09	219/403	20/46	0.83 (0.47–1.47)	0.515	
>30.09	268/164	22/27	0.45 (0.24–0.84)	**0.013**	
Pre-BMI (Kg/m^2^)					0.050
≤22.23	214/385	19/48	0.72 (0.41–1.30)	0.281	
>22.23	272/181	23/25	0.52 (0.28–0.98)	**0.042**	
SBP (mmHg)					0.380
≤110.08	235/324	20/48	0.55 (0.31–0.98)	**0.043**	
>110.08	252/243	22/25	0.73 (0.38–1.43)	0.363	
DBP (mmHg)					**0.010**
≤69.53	239/301	21/24	0.57 (0.32–1.03)	0.062	
>69.53	248/266	21/32	0.67 (0.35–1.27)	0.218	
FPG (mmol/L)					**<0.001**
≤4.79	194/498	12/67	0.40 (0.20–0.78)	**0.007**	
>4.79	293/69	30/6	1.12 (0.44–2.88)	0.814	
1 h PG (mmol/L)					0.050
≤8.26	111/445	10/62	0.57 (0.28–1.18)	0.129	
>8.26	376/122	32/11	0.94 (0.45–1.96)	0.861	
2 h PG (mmol/L)					0.873
≤7.11	140/467	10/63	0.47 (0.23–0.98)	**0.043**	
>7.11	347/100	32/10	0.88 (0.41–1.89)	0.742	
HbA1c (%)					**<0.001**
≤5.20	176/419	13/56	0.51 (0.27–0.97)	**0.04**	
>5.20	311/148	29/17	0.75 (0.38–1.48)	0.412	
TG (mmol/L)					0.137
≤2.54	251/355	24/46	0.64 (0.36–1.12)	0.118	
>2.54	236/212	18/27	0.60 (0.31–1.17)	0.134	

a, Variables’ stratification was based on their mean value, respectively; b, Adjusted for age, pre-BMI in logistic regression models; c, Test for multiplicative interaction obtained from logistic regression models. Bold values indicate that the differences are statistically significant.

### 3.3 FPRP analysis

Under the preset threshold value of 0.2 and a prior probability of 0.1, the FPRP analysis was used to evaluate the noteworthy associations between rs1595066 and GDM risk. However, the only association between rs1595066 and GDM risk in subjects with FPG ≤4.79 mmol/L seems to be genuine (*P*_FPRP_ = 0.199), and the other positive associations observed may be obtained by chance and should be accepted cautiously, as shown in **Table 4**.

**Table 4:** FPRP analysis for the positive associations of rs1595066 and GDM risk.

Comparison	Adjusted OR (95% CI)	*P*	Statistical Power	Prior probability					
				0.25	0.1	0.01	0.001	0.0001	0.00001
rs1595066									
TT vs. CC	0.60 (0.38–0.94)	0.026	0.788	0.090	0.229	0.766	0.971	0.997	1.000
TT vs. CC/CT	0.61 (0.40–0.95)	0.027	0.804	0.092	0.232	0.769	0.971	0.997	1.000
Subgroup									
Age >30.09 (year)	0.45 (0.24–0.84)	0.013	0.379	0.093	0.236	0.772	0.972	0.997	1.000
BMI >22.23 (Kg/m^2^)	0.52 (0.28–0.98)	0.042	0.544	0.188	0.410	0.884	0.987	0.999	1.000
SBP ≤110.08 (mmHg)	0.55 (0.31–0.98)	0.043	0.629	0.170	0.381	0.871	0.986	0.999	1.000
FPG ≤4.79 (mmol/L)	0.40 (0.20–0.78)	0.007	0.254	0.076	**0.199**	0.732	0.965	0.996	1.000
2hPG ≤7.11 (mmol/L)	0.47 (0.23–0.98)	0.043	0.431	0.231	0.473	0.908	0.990	0.999	1.000
HbA1C ≤5.20 (%)	0.51 (0.27–0.97)	0.040	0.524	0.186	0.407	0.883	0.987	0.999	1.000

Bold values indicate that the differences are statistically significant.

### 3.4 MDR analysis for variants interactions

Under the recessive genetic model of variants, MDR analysis revealed that rs1595066 was the best single-locus model for GDM risk (TBA: 0.5226, CVC: 100/100, *P<*0.0001). The best multi-loci model was the 4-factor model including rs1595064, rs1595065, rs1595066, and rs6719645 (TBA: 0.5494, CVC: 100/100, *P* = 0.0006) (**Table 5**).

**Table 5:** MDR analysis for *ERBB4* SNPs to predict GDM risk.

Best model	Training balanced accuracy	Testing balanced accuracy	CVC	χ^2^	*P*
1	0.5226	0.5226	100/100	29.54	<0.0001
1, 2	0.5406	0.5369	99/100	22.74	<0.0001
1, 3, 4	0.5457	0.5457	100/100	10.49	0.0012
1, 2, 3, 4	0.5494	0.5494	100/100	11.88	0.0006

CVC, cross validation consistency; 1, rs rs1595066; 2, rs1595064; 3, rs1595065; 4, rs6719645.

### 3.5 Functional analysis by computer tool

As rs1595066 is a 3′ UTR variant located at the microRNA binding site, functional analysis by SNP function prediction (FuncPred) in the SNPinfo Web Server was performed in our study. The predictive results indicated that *ERBB4* was regulated by a series of miRNAs (**Table 6**). The minimum free energy of hybridization (MFE) was altered in hsa-miR-221, hsa-miR-520g, and hsa-miR-520h when the allele of the mRNA forward sequence changed from C to T. Meanwhile, it seems that allelic status can disrupt miRNA binding sites, causing miRNAs (hsa-miR-548l, hsa-miR-106a, hsa-miR-17, and hsa-miR-20a) that bind to specific sequences of *ERBB4* to no longer bind. The above findings revealed that rs1595066 may change individuals′ susceptibility to GDM by disturbing miRNA binding to *ERBB4* mRNA.

**Table 6:** Prediction for miRNA binding sites and altered MFE by rs1595066.

Allele	Position	Strand	Forward sequence	hsa-miRNA	Score	MFE
C	8	–	cAAAGTA *C* CTTACTTTAA AAGAAc	hsa-miR-106a	145	−11.25
C	8	–	CAAAGTA *C* CTTACTTTA AAAGAAc	hsa-miR-17	150	−15.49
C	8	–	CAAAGTA *C* CTTACTTTAA AAGAAc	hsa-miR-20a	142	−11.63
T	20	–	tGCTACATATTTCAAAGTA *T* C	hsa-miR-221	151	−11.36
C	20	–	tGCTACATATTTCAAAGTA *C* C	hsa-miR-221	147	−11.2
C	18	–	CTACATATTTCAAAGTA *C* CTT	hsa-miR-448	141	−11.85
C	6	–	AAGTA *C* CTTACTTTAAAA gaac	hsa-miR-515-3p	151	−18.36
C	8	–	CAAAGTA *C* CTTACTTT AAAAga	hsa-miR-519d	165	−22.47
C	6	–	AAGTA *C* CTTACTTTAAAA gaac	hsa-miR-519e	151	−16.74
C	7	–	AAAGTA *C* CTTACTTTAAA AGA	hsa-miR-520b	146	−17.91
C	7	–	AAAGTA *C* CTTACTTTAAA AGAa	hsa-miR-520c-3p	146	−18.23
C	6	–	AAGTA *C* CTTACTTTAAAA GAac	hsa-miR-520f	141	−17.2
T	9	–	tCAAAGTA *T* CTTACTTTAA AAgaa	hsa-miR-520g	153	−14.57
C	9	–	tCAAAGTA *C* CTTACTTTAA AAgaa	hsa-miR-520g	161	−18.96
T	9	–	tCAAAGTA *T* CTTACTTTAA AAg	hsa-miR-520h	153	−14.57
C	9	–	tCAAAGTA *C* CTTACTTTAA AAg	hsa-miR-520h	161	−18.96
C	8	–	cAAAGTA *C* CTTACTTTAaa	hsa-miR-548k	148	−11.92
T	8	–	cAAAGTA *T* CTTACtttaaaagaa	hsa-miR-548l	147	−8.75
C	8	–	CAAAGTA *C* CTTACTTTAA AAGAA	hsa-miR-93	140	−12.48

MFE, minimum free energy.

### 3.6 Construction and validation of the nomogram prediction model

**Figure 3:**
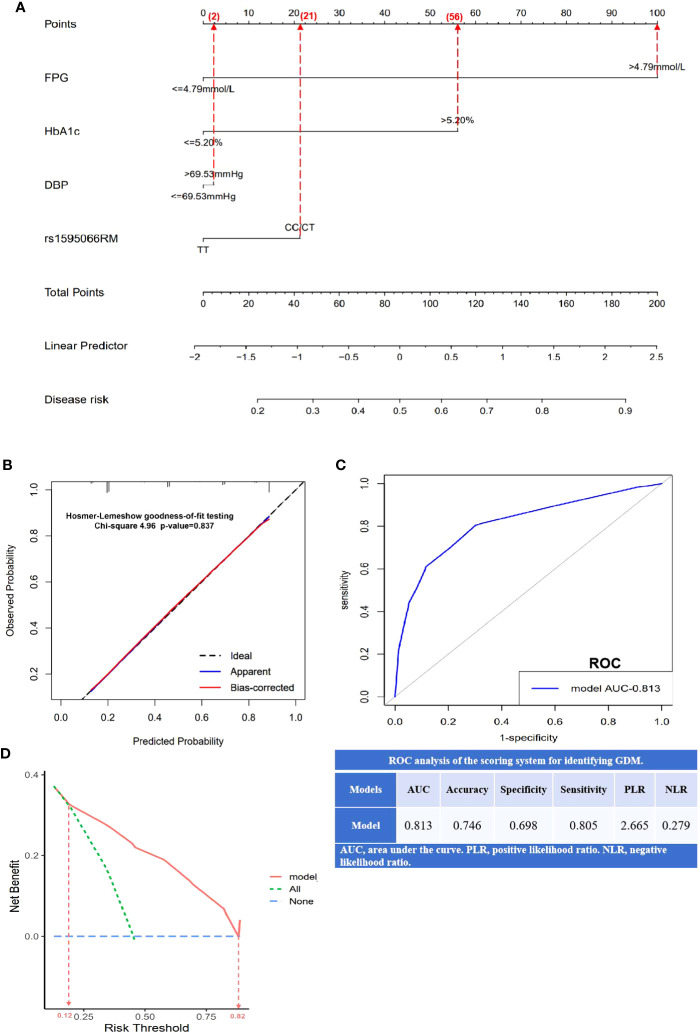
Establishment and validation of nomogram model for predicting GDM risk. **(A)** A nomogram model constructed using FPG, HbA1c, DBP, and rs1595066, based on binary logistic regression. The value of each variable was scored on a point scale from 0 to 100, after which the scores for each variable were added. That sum is located on the total points axis, which enables us to predict the probability of GDM risk; **(B)** the calibration curve was evaluated by Hosmer–Lemeshow goodness-of-fit testing with a *p*-value of 0.837; **(C)** the area under the receiver operating characteristic curve (ROC) and the scoring system for identifying GDM; **(D)** the decision curve analysis (DCA) with higher clinical bet benefit in probability threshold in 0.12 to 0.82.

This study used DBP, FPG, HbA1c, and the recessive model of rs1595066 as independent predictive factors to construct a nomogram for GDM prediction (**Figure 3A**). Based on the scores of the four factors, the total score of the prediction model was obtained, and a high total score indicated a high risk of developing GDM. As shown in **Figure 3B**, the calibration curve was validated to be almost coincident with the ideal line, indicating a good consistency between the predicted probability and the actual observed probability with a *P*-value of 0.837. The area under the ROC curve (AUC) was 0.813, with a sensitivity of 80.5% and a specificity of 69.8%, demonstrating a well-accepted predictive and discriminative performance (**Figure 3C**). As shown in DCA (**Figure 3D**), when the risk threshold was between 0.12 and 0.82, that predictive nomogram model will provide higher clinical net benefit compared with the “treat all” or “treat none” strategies.

## 4 Discussion

GDM can cause adverse health events in both mothers and babies in the short- and long-term. It is an independent risk factor for the long-term risk of T2DM, metabolic syndrome, cardiovascular disease, multiple tumors, and kidney disease in mothers. Offspring may increase the risk of adverse health consequences, such as T2DM, obesity, impaired neurodevelopmental outcomes, and even eye diseases. Studies have confirmed that GDM and T2DM are both multifactorial diseases and share some epidemiological risk factors, such as obesity, insulin resistance, impairment of β-cell function, race, medical history, and family history (34, 35). GDM is affected by numerous environmental exposure factors, susceptibility genes, genetic variations, and even their gene–gene and gene–environment interaction effects, and/or effect modifications.

Currently, candidate gene studies and GWAS have identified many genetic susceptibility variants for GDM. A GWAS by Cho et al. investigated numerous SNPs known to be associated with an increased risk of GDM in Koreans (36). Our previous candidate gene association studies have also confirmed that angiotensin-converting enzyme 2 (*ACE-2*), retinoic acid X receptor-γ (*RXR-γ*), CDK5 regulatory subunit-associated protein 1-like 1 (*CDKAL1*), melatonin receptor 1 B (*MTNR1B*), and transcription factor 7 analog-2 (*TCF7L2*), which either affect GDM risk or are significantly associated with genetic susceptibility to GDM through interactions with age, pre-BMI, and blood lipids (12–15). ERBB4 activates multiple downstream signaling pathways by binding to specific ligands, participating in the regulation of insulin sensitivity and glucose intake, and maintaining energy balance in the body (20–22). Studies have shown that the *ERBB4* variant rs7588550 can be significantly associated with the risk of type 1 and type 2 diabetic nephropathy by affecting the *ERBB4* expression (23, 24). However, there have been no studies on the relationship between *ERBB4* variants and GDM risk.

In the present study, four functional tag SNPs were genotyped to explore the genetic susceptibility of *ERBB4* variants to GDM. Significant effects of rs1595066 on the risk of GDM were found in the Guilin population of China, especially in subjects over 30.09 years old, with BMI ≥22.23 Kg/m^2^, SBP higher than 110.08 mmHg, etc. This suggests that *ERBB4* variants could also alter an individual’s susceptibility to GDM by modulating key physiological and biochemical variables within the body. In addition, MDR analysis also confirmed that rs1595066 is significantly associated with the risk of GDM and is the best one-factor model for predicting GDM risk. Meanwhile, a complex gene–gene combination effect was also detected, and all four studied SNPs made the best multi-loci model for predicting GDM risk. This finding indicates that personal differences suffering in GDM may be affected by SNP–SNP or SNP–environment interactions. Based on this, the nomogram model established by the environmental factors and genetic variants (FPG, DBP, HAb1c, and rs1595066) demonstrated good calibration and discrimination ability, and DCA also showed promisingly clinical application value. This predictive model will be helpful for individuals to prevent GDM early in pregnancy.

MicroRNAs (miRNAs) can bind to specific sites in the 3′-UTR of target mRNA, playing a vital role in gene expression regulation, while SNPs of target gene miRNA-binding sites may alter the binding affinity of potential miRNAs to target mRNA (37). Thus, changes in the stability of miRNA–mRNA combinations are likely to have a profound impact on the transcriptional and post-transcriptional regulation of gene expression (38, 39). Studies have suggested that the *PTPRD* gene miR-450a binding site SNP rs56407701 can significantly affect the susceptibility of Chinese Han women to GDM during pregnancy by regulating *PTPRD* expression (40), while the miR-323b-5p-binding site rs1063192 in the *CDKN2B* gene was significantly associated with GDM. Further analysis showed that subjects with the CC genotype exhibited increased glucose levels at 1 h and 3 h, higher insulin levels at 3 h during an OGTT, as well as lower TC and LDL-c levels compared with TT genotype carriers. In the present study, from the *in silico* analysis, we can see that rs1595066 is located at *ERBB4* gene miRNA binding sites, and seems to have the effect of creating a new or disrupting an existing binding site, or leading to significant changes in miRNA–mRNA binding minimum free energy (MFE) under different alleles (41). Thus, the miRNA-binding site SNP may play multiple roles in regulating the body’s genetic susceptibility to complex human diseases.

This study had some limitations. First, this was a hospital-based case–control study. Therefore, there will inevitably be a bias in research object selection and retrospective data bias. Second, potential confounding factors of GDM, such as smoking status, poor obstetrics, malnutrition, and socioeconomic factors, were not assessed. They are likely to interfere with the objectivity of the associations and strengths between the studied *ERBB4* variants and GDM onset. Third, although a relatively large sample study design was adopted in this study, the very low frequency of genotypes tested in the studied variants may still limit the statistical performance, especially in subgroup analysis. Finally, this study only explored the potential biological functions of positively associated variants using bioinformatics tools, but molecular experimental research has not been conducted.

## 5 Conclusion

*ERBB4* variants, such as rs1595066, were significantly associated with the onset of GDM. Interactions between variants or variants-clinical variables and the binding stability of miRNAs to target *ERBB4* gene sequence changing may be the mechanisms by which *ERBB4* gene variants affect the susceptibility of pregnant women to GDM. This nomogram model will be useful for the early prevention of GDM.

## Data availability statement

The data presented in the study are deposited in Dryad repository, accession number https://doi.org/10.5061/dryad.qz612jmnr.

## Ethics statement

The studies involving humans were approved by The Ethics Committee of Guilin Medical University. The studies were conducted in accordance with the local legislation and institutional requirements. The participants provided their written informed consent to participate in this study.

## Author contributions

RL: Data curation, Formal analysis, Writing – original draft. YW: Data curation, Formal analysis, Writing – original draft. LY: Data curation, Formal analysis, Writing – original draft. PZ: Data curation, Writing – review & editing. GH: Software, Writing – review & editing. QL: Methodology, Supervision, Visualization, Writing – review & editing. XY: Conceptualization, Project administration, Funding acquisition, Writing – review & editing.
